# CrAssphage as a Human Enteric Viral Contamination Bioindicator in Marketed Bivalve Mollusks

**DOI:** 10.3390/v17071012

**Published:** 2025-07-18

**Authors:** Isabella Rodrigues Negreiros, Natália Lourenço dos Santos, Bruna Barbosa de Paula, Bruna Lopes Figueiredo, Marcelo Luiz Lima Brandão, José Paulo Gagliardi Leite, Marize Pereira Miagostovich, Carina Pacheco Cantelli

**Affiliations:** 1 Laboratory of Comparative and Environmental Virology, Oswaldo Cruz Institute/Fiocruz, Rio de Janeiro 21040-900, Brazil; 2 Department of Experimental and Preclinical Development, Bio-Manguinhos/Fiocruz, Rio de Janeiro 21040-900, Brazil

**Keywords:** CrAssphage, enteric viruses, fecal contamination, bivalve mollusks, food safety, sanitary surveillance

## Abstract

CrAssphage, a bacteriophage that infects human gut-associated *Bacteroides* spp., has emerged as a potential anthropogenic fecal pollution indicator in environmental matrices. This study investigated the presence and concentration of crAssphages in bivalve mollusks (oysters and mussels) marketed in three cities in the state of Rio de Janeiro, Brazil, sampled from January to December 2022. CrAssphages were detected during the study period in 66.7% (48/72) of sampled oysters and 54.8% (34/62) of sampled mussels, at median concentrations of 1.9 × 10^4^ and 4.2 × 10^4^ genome copies (GC)/g, respectively. These levels were 1–2 log_10_ higher than those observed for major human enteric viruses, including norovirus genogroups GI and GII, sapovirus, human mastadenovirus (HAdV), rotavirus A, human astrovirus (HAstV), and hepatitis A virus. CrAssphage specificity and sensitivity were calculated for all viruses. Moderate correlations between crAssphage (log_10_ GC/g) and norovirus GI and GII, HAdV, SaV, and HAstV (Spearman’s *rho* = 0.581–0.464, *p* < 0.001) were observed in mussels. Altogether, the data support the use of crAssphage as a molecular indicator of human viral contamination in shellfish, with potential application in routine environmental and food safety monitoring in production areas.

## 1. Introduction

The global consumption of aquatic food products has increased substantially in the last decades and is projected to continue its upward trend in the coming years. According to the Food and Agriculture Organization of the United Nations (FAO), marine animal production is expected to increase by an additional 17%, reaching 205 million tons in 2032 [1]. However, seafood spoilage and quality degradation comprise critical challenges, resulting in the loss of valuable nutritional components and raising significant food safety concerns [2]. In response, the FAO and the World Health Organization (WHO) have issued guidelines for Bivalve Mollusk Sanitation Programs focusing on the management of production areas [3]. These guidelines, based on the *Codex Alimentarius* standard for live and raw bivalve mollusks [4], require that live bivalve mollusks used for direct human consumption meet specific *Escherichia coli* limits by the MPN method according to the ISO 16649-3 standard or an equivalent guideline. In Brazil, the Ministry of Agriculture and Livestock has implemented the National Safe Bivalve Mollusk Program (MoluBis Program/MAPA Regulation 884/2023), which establishes measures for the hygienic-sanitary control and inspection of bivalve mollusks intended for human or animal consumption [5], following the same parameters. However, bacteria are less resistant and persistent to wastewater treatment compared to enteric viruses [6,7], making them ineffective as viral infection risk indicators. In this sense, the use of only one fecal indicator bacterium in bivalve monitoring programs neglects the role of bivalves as a vehicle for the transmission of enteric viruses, such as norovirus, known for its role in foodborne disease outbreaks [8,9].

In 2023, while reviewing scientific evidence based on frequency and severity criteria, the Joint FAO/WHO Expert Meeting on Microbiological Risk Assessment (JEMRA) highlighted the importance of additional research to determine an appropriate viral indicator for use in food commodities associated with foodborne virus contamination [10]. In this respect, several viral contamination indicators, including bacteriophages (F-specific RNA bacteriophage genogroup II, F-RNA phage II) [11] and plant viruses (pepper mild mottle virus, PPMMoV) [12,13], as well as human (mastadenovirus and JC polyomavirus) and animal viruses (bovine polyomavirus) [14], have been studied in environmental waters.

More recently, crAssphage (cross-Assembly phage) has emerged as a promising human faecal contamination indicator [15,16], as it is one of the most ubiquitous human gut viruses, with *Bacteroides* species as the putative hosts [17,18]. CrAssphage is a double-stranded DNA genome phage containing 97 kilobases (kb), with a tail and icosahedral 77–88 nm capsid [19], widely prevalent in human populations from different geographical areas, including the United States of America (USA), Europe, Africa, and Asia [20]. Moreover, variations in the abundance of these markers are noted in different regions, with lower detection levels observed in Africa and Asia [21,22,23]. This evidence suggests that crAssphage investigations in new geographical areas require validation based on local samples.

This study aimed to investigate the detection and concentration of crAssphages in marketed bivalves, as well as potential correlations with the simultaneous presence of enteric viruses previously analyzed in these samples, including norovirus, sapovirus (SaV), human mastadenovirus (HAdV), rotavirus A (RVA), human astrovirus (HAstV), and hepatitis A virus (HAV) [24,25]. This study provides the first scientific evidence of crAssphage detection and quantification in bivalve mollusks intended for human consumption in Brazil and, more broadly, across Latin America.

## 2. Material and Methods

This study investigated crAssphage occurrence and concentrations in mussels (*Perna perna* and *Perna viridis*) and oysters (*Crassostrea gigas* and *Crassostrea gasar*) marketed in three cities in the state of Rio de Janeiro, Brazil (Angra dos Reis, Rio de Janeiro, and Niterói) acquired from January to December 2022. The oysters purchased in Niterói and Rio de Janeiro were farmed at two production sites in the city of Florianópolis, in the state of Santa Catarina (SC), Southeastern Brazil. Mussels were obtained from one mussel farmer at Angra dos Reis, and two commercial points in Niterói and Rio de Janeiro. Samples were processed according to the ISO 15216-1:2017 [26]. Ten microliters of internal process control, MgV vMC_0_ (7.7 × 10^6^ genome copies (GC)/µL), were spiked in each bivalve sample (a pool of 12–15 digestive glands was dissected) and homogenized for 3 min in a vortex with 2 mL of a proteinase K solution (100 µg/mL). This mixture was incubated at 37 °C with shaking (250 rpm) for 70 min, followed by incubation at 60 °C for 20 min, and centrifugation at 3000× *g* for 5 min was performed. The supernatants recovered were immediately processed or stored at −80 °C. The presence, concentration, and characterization of norovirus GI/GII, RVA, SaV, HAdV, HAstV, and HAV were determined as described previously [24,25]. Total nucleic acid extracted was carried out employing the silica-magnetic bead extraction method using the MDX^®^ DNA and RNA Pathogens kit from Extracta Loccus^®^ (Biotech Research Supplies, Rio de Janeiro, Brazil), according to the manufacturer’s instructions.

### 2.1. CrAssphage Quantification

CrAssphage quantification was performed using a TaqMan^®^ qPCR System (ABI PRISM 7500^®^, Applied Biosystems, Foster City, CA, USA) with a set of CPQ_056 primers (forward 056F1, 5′-CAG AAG TAC AAA CTC CTA AAA AAC GTA GAG-3′; reverse 056R1, 5′-GAT GAC CAA TAA ACA AGC CAT TAG C-3′), and probe 056P1 (5′-HEX-AAT AAC GAT TTA CGT GAT GTA AC-MGB-3′) as previously described by Stachler et al. (2017) [27]. Molecular reactions were performed using the TaqMan^TM^ Universal Master Mix kit (Applied Biosystems, Foster City, CA, USA) in a 20 µL reaction mix containing 12.5 pmol of each primer, 7.5 pmol of the probe, and 5 µL of viral nucleic acid. Positive controls (containing DNA extracted from fecal human suspensions), negative controls (DNAse and RNAse-free water), and no template controls (NTC) were included in all qPCR assays. To check for possible inhibitors in the qPCR tests, the isolated nucleic acids obtained from each bivalve sample were tested pure (undiluted) and 10-fold diluted, both in duplicate. Standard crAssphage curves were generated using 10-fold serial dilutions of a double-stranded DNA fragment containing the crAssphage genome amplification region sequence from position 14,731 nucleotides (nt) to 14,856 nt, ORF0024 region (gBlock Gene Fragment, Integrated DNA Technologies^®^, Coralville, IA, USA). Amplification was carried out by applying the following thermal cycling conditions: a hold step at 95 °C for 2 min, followed by 45 cycles at 95 °C for 15 s, and 60 °C for 60 s. The standard curves indicated slopes ranging from −3.286 to −3.448, and square regression coefficient (r^2^) values varying between 0.995 and 1.000, indicating high reaction efficiencies from 94.9 to 101.5%. Samples were considered positive when at least one analyzed duplicate (pure or 10-fold diluted) was tested positive at a cycle threshold (Ct) < 40 presenting a characteristic sigmoid curve.

### 2.2. CrAssphage as a Marker for Each Evaluated Enteric Virus

The performance of the crAssphage marker indicator was calculated for each analyzed enteric virus according to the equations described by Suh et al. (2024) [28], where sensitivity was set as the amount of positive samples in which the crAssphage was detected, while specificity comprised the amount of negative samples in which the crAssphage was not detected [29]. The following formulas [30] were applied: sensitivity = TP/(TP + FN), and specificity = TN/(TN/FP), where the true positive (TP) refers to the number of enteric virus-positive samples, and false negative (FN) refers to the number of enteric virus-positive and crAssphage-negative samples. True negative (TN) refers to the number of enteric virus-negative samples, whereas false positive (FP) refers to the number of enteric virus-negative and crAssphage-positive samples.

### 2.3. Statistical Analyses

Viral concentrations in the DNA GC/g recovered from the bivalve samples were analyzed for significant differences by applying the independent sample Mann–Whitney U Test using the GraphPad Prism version 9.0.0^®^ software (GraphPad Software^®^, San Diego, CA, USA). Data were checked for normality by the Shapiro-Wilk normality test. Subsequently, non-parametric tests (Kruskal-Wallis test followed by Dunn’s multiple comparisons post-test) were carried out for the results obtained across the different cities assessed herein and a Pearson’s linear correlation analysis was applied for enteric virus viral and crAssphage (log_10_ GC/g) concentrations using the Jamovi^®^ version 2.6.24 software [31] for each bivalve species. Box-and-whisker plots were plotted to indicate differences between medians. Results were considered statistically significant at *p* < 0.05.

## 3. Results and Discussion

### 3.1. CrAssphage Detection

**Figure 1:**
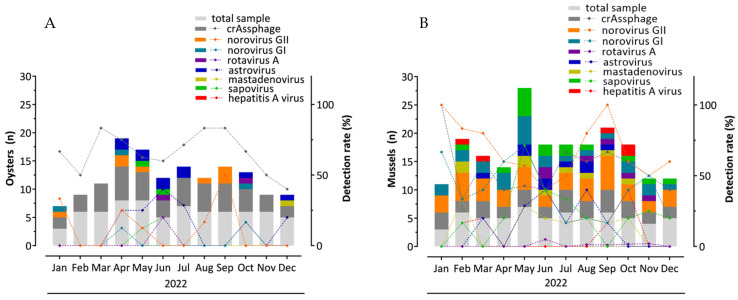
CrAssphage and enteric virus distributions and percentages in marketed bivalve mollusks (oysters (**A**) and mussels (**B**)) sampled over 12 months (from January to December 2022) in Angra dos Reis, Niterói, and Rio de Janeiro, Brazil.

The primary aim of this study was to demonstrate the applicability of crAssphage as a human fecal contamination viral indicator in commercially sourced oysters and mussels. The findings revealed that the crAssphage occurred year-round in the investigated oyster and mussel samples, presenting total detection rates of 66.7% (48/72) and 54.8% (34/62), respectively (Table 1 and Figure 1). This corroborates other studies where crAssphage was detected in sediment and mussel samples (62–75%) [32], oysters (50% and 70%) [33,34], processing water (50%) [35], irrigation water (61%), fresh leafy greens (68.5%), and stream water (58.5%) [28]. Additionally, several studies have demonstrated its widespread presence in wastewater and various fecal-contaminated water bodies at consistently high concentrations and displaying minimal seasonal fluctuations [32,36,37].

Metagenomic sewage sample analyses have revealed a significantly higher CrAssphage abundance compared to other viral markers such as PMMoV, HAdV-F, and Human Polyomavirus BK (HPyV) [21]. The use of a specific set of primers and probes, such as the CPQ_056 and CPQ_064, has been recognized for its robust performance in monitoring human-associated viral contamination, making it a reliable environmental surveillance tool [27,28,38].

**Figure 2:**
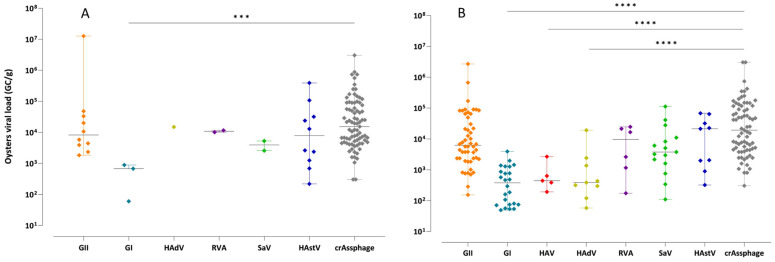
CrAssphage and enteric virus loads [genome copies per gram (GC/g) of digestive tissue] in oysters (**A**) and mussels (**B**) marketed over 12 months (from January to December 2022). Box and whisker plots depict all values distributed within the median (horizontal line in the box) and the range of concentrations (GC/g) detected during the sampling period. **** *p* < 0.001; *** *p* < 0.01.

**Figure 3:**
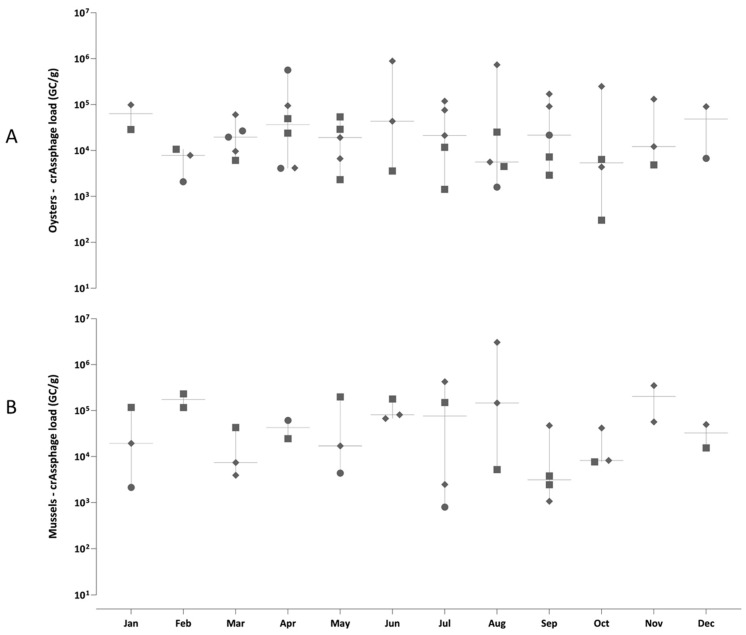
CrAssphage loads [genome copies per gram (GC/g) of digestive tissue] detected in oysters (**A**) and mussels (**B**) sampled over 12 months (from January to December 2022) in Angra dos Reis (circle), Niterói (square), and Rio de Janeiro (diamond), Brazil. Box and whisker plots depict all values distributed within the median (horizontal line in the box) and the range of concentrations (GC/g) detected during the sampling period.

CrAssphage concentrations ranged from 5.4 × 10^3^ to 6.4 × 10^4^ GC/g [1.9 × 10^4^ GC/g (total median value)] in oysters (Figure 2A and Figure 3A), and from 3.1 × 10^3^ to 2.0 × 10^5^ GC/g [4.2 × 10^4^ GC/g (total median value)] in mussels (Figure 2B and Figure 3B). These values were 1–2 log_10_ higher than enteric virus concentrations (norovirus, SaV, HAdV, RVA, HAstV, and HAV) reported by other studies for different matrices [28,32]. Statistically significant differences were observed for crAssphage compared to norovirus GI, HAV, HAdV (*p* < 0.001) (Figure 2B), SaV (*p* < 0.01), and norovirus GII (*p* < 0.05) in mussels when assessing the viral loads between all the investigated enteric viruses. Bacterial and viral contamination of water poses significant public health risks due to diverse pathogens causing a range of illnesses. Given the limitations of bacterial indicators in detecting enteric viruses, crAssphage has emerged as a reliable human-specific marker owing to its abundance, specificity, and persistence [15,27].

**Figure 4:**
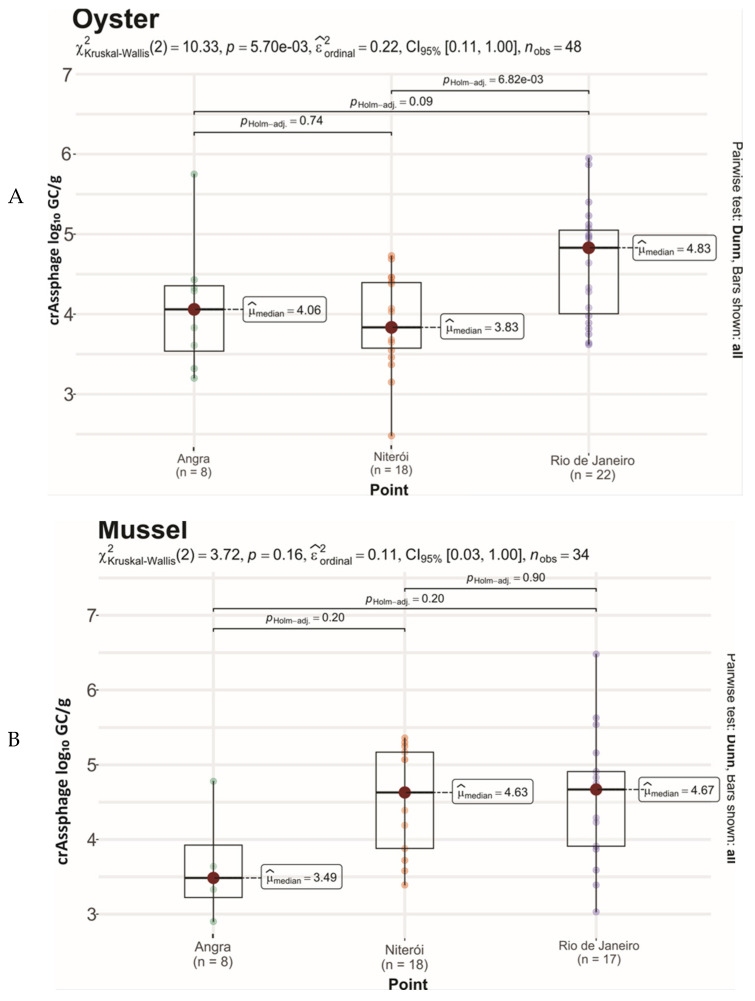
CrAssphage loads [log_10_ genome copies per gram (GC/g) of digestive tissue] in oysters (**A**) and mussels (**B**) marketed over 12 months (from January to December 2022) in Angra dos Reis, Niterói, and Rio de Janeiro, Brazil. Box and whisker plots depict all values distributed within the median (horizontal line in the box) and the range of concentrations (log_10_ GC/g) detected during the sampling period.

A significantly higher crAssphage concentration was detected in oyster samples from Rio de Janeiro (4.83 log_10_ GC/g) compared to Niterói (3.83 log_10_ GC/g, *p* < 0.01) (Figure 4). This may be associated with differences concerning their source, as they were obtained from distinct aquaculture farms in Florianópolis, in the state of Santa Catarina. This state has a 561 km coastline formed by sheltered bays and numerous islands, and is the most important shellfish production zone in Brazil, representing nearly 95% of all national shellfish production [39,40]. Gyawalli et al. (2021) [34] observed similar crAssphage concentrations (3.84 ± 0.41 log_10_ GC/g) in oyster samples collected in 2019 from one river and two different commercial oyster farms in New Zealand. In another study, Venuti et al. (2025) [33] reported a mean crAssphage concentration of 3.72 log_10_ GC/g in Mediterranean mussels (*Mytilus galloprovincialis*) obtained from local retail stores in the Campania region (Southern Italy) in April and June 2023.

Geographic variations may have affected virus detections in both analyzed bivalve species investigated. Samples from Rio de Janeiro and Niterói exhibited the highest crAssphage prevalence (87.5 and 79.5%, respectively) compared to Angra dos Reis (24%) (Table 1), corroborating studies reporting that crAssphage concentrations are dependent on the human populations served by wastewater treatment plants [32,38,41]. In accordance, the data reported herein may be associated to the estimated populations of the sampled cities [(6.7 million—Rio de Janeiro (collected mussels); 576,361—Florianópolis (oysters farmed and marketed in Rio de Janeiro and Niterói); 516,720—Niterói (collected mussels); and 179,120—Angra dos Reis (farmed and marketed oysters and mussels)] [42]. Furthermore, studies examining crAsssphage phylogeography have reported that industrialization levels may influence its abundance in the human gut microbiome [43,44]. Corroborating this, Angra dos Reis displays the lowest industrialization levels compared to the other sampled cities.

### 3.2. Evaluation of crAssphage as an Enteric Virus Detection Marker

Sensitivity and specificity values were calculated for each evaluated enteric virus to determine how crAssphage could act as a viral marker. According to Table 2, crAssphage presented a high specificity value for norovirus GII/GI (0.9 and 0.8), and 0.7 for other enteric viruses in mussels, and 0.7 for HAV in oysters. High sensitivity values (1.0) were observed for SaV and HAstV in both bivalves, as well as RVA, HAV, and HAdV in mussels. Suh et al. (2024) [28], when determining the prevalence and abundance of crAssphages in different matrices, reported crAssphage specificity and sensitivity values for norovirus ranging from 0.56 to 0.68, and from 0.75 to 0.71 in fresh leafy greens, irrigation water, and stream water, respectively.

According to the Spearman analysis, crAssphage was moderately correlated among viral titers (log_10_ GC/g) with norovirus GII (*r* = 0.536, *p* < 0.001), norovirus GI (*r* = 0.580, *p* < 0.001), HAdV (*r* = 0.485, *p* < 0.001), SaV (*r* = 0.487, *p* < 0.001), and HAstV (*r* = 0.463, *p* < 0.001) in mussels, while a weak correlation with HAstV (*r* = 0.388, *p* < 0.001) was observed for oysters (Table 3). Previous studies have reported higher crAssphage abundances and correlations with enteric viruses compared to other faecal markers, supporting its use as a human-specific source tracking marker in environmental matrices like sewage, rivers, and food [28,32,45,46,47]. Wu et al. (2025) [48], for example, reported a higher crAssphage correlation with adenovirus (*r* = 0.64) in secondary treated effluents from California, Florida, and Ohio (USA). Other studies have also reported correlations between adenovirus and crAssphage in untreated wastewater (r = 0.51) [49], and environmental water sediment (*r* = 0.37) [32]. Interestingly, Farkas et al. (2019) [32] reported a strong correlation between crAssphage and JC polyomavirus (human virus [14]) in wastewater effluent (*r* = 0.67, *p* < 0.001). Recently, Venuti et al. (2025) [33] demonstrated strong correlations (ρ > 0.65, *p* < 0.05) between crAssphage and norovirus GII, HAstV, and RVA concentrations in mussels (*Mytilus galloprovincialis*) marketed in Southern Italy.

Studies have demonstrated the prevalence and environmental stability of crAssphage compared to other viral microbial source tracking (MST) candidates [17,50]. It is consistently detected at high concentrations in untreated wastewater and shows a strong correlation with human fecal contamination, outperforming traditional MST markers in both sensitivity and specificity [32,51,52,53,54]. Although low-level detection in livestock-associated matrices has been reported, crAssphage remains predominantly associated with human sources [27,38,41,55]. Its persistence and physicochemical resilience in diverse aquatic environments further support its application in long-term water quality surveillance [56].

A recent multicontinental study by Toribio-Avedillo et al. (2025) [57] evaluated the performance of crAssphage, HMBif (a human-specific *Bifidobacteria* marker), and HF183 (a human-associated *Bacteroidales* marker) across diverse environmental matrices, including wastewater influents, effluents, and riverine samples from Europe, Asia, and Africa. CrAssphage consistently exhibited the highest detection frequencies and concentrations across all regions and sample types. These findings support its global applicability as a robust viral indicator of human fecal pollution and highlight the relevance of geographically tailored MST strategies in environmental monitoring programs.

This is the first documented evidence of crAssphage detection and quantification in marketed bivalve mollusks for human consumption in Brazil and, more broadly, in Latin America, highlighting a relevant contribution to the regional understanding of viral contamination in shellfish.

## 4. Conclusions

The results confirm the successful integration of crAssphage quantification into microbial monitoring frameworks, establishing its utility as a complementary tool to enhance food safety assessments along bivalve supply chains. This molecular approach enables more comprehensive and targeted risk assessments, supports cost-effective monitoring strategies through optimized resource allocation, and reinforces public health protection measures.

The routine application of crAssphage as an MST marker improves the sensitivity and specificity of human fecal contamination detection in aquatic environments, strengthening sanitary surveillance programs for both aquaculture and marketed bivalves. To expand its applicability across diverse environmental contexts, subsequent studies should include samples from multiple Brazilian regions and incorporate comparative analyses with other viral indicators and crAssphage genomic targets to evaluate marker performance under varying ecological conditions.

## Figures and Tables

**Table 1:** CrAssphage detection rates (per bivalve species/city, and total per species) in bivalve mollusks acquired between January and December 2022 from three commercial sites in the state of Rio de Janeiro, Brazil.

Commercial Site	Bivalve	Origin/Farm–City	*n*	CrAssphage Detection (%)		
				Per Species/Site	*p*-Value	Total/Site
Angra dos Reis	Oyster	Ilha Grande Bay, Angra dos Reis (same farm)	25	8 (32.0)	0.0109	12/50 (24.0)
	Mussel		25	4 (16.0)		
Niterói	Oyster	Florianópolis, farm X	25	22 (88)	0.0646	35/40 (87.5)
	Mussel	Sampled from any point in Niterói	15	13 (86.7)		
Rio de Janeiro	Oyster	Florianópolis, farm Y	22	18 (81.8)	0.0121	35/44 (79.5)
	Mussel	Sampled from any point in Rio de Janeiro	22	17 (77.3)		

**Table 2:** Sensitivity and specificity values for crAssphage employed as an enteric virus marker in oyster and mussel samples commercially acquired and collected between January and December 2022.

Bivalve	Parameter	Viruses						
		Norovirus GII	Norovirus GI	Sapovirus	Astrovirus	Rotavirus A	Mastadenovirus	Hepatitis A Virus
Oyster	Sensitivity	0.7	0.6	1.0	1.0	1.0	0.5	-
	Specificity	0.6	0.4	0.4	0.6	0.6	0.6	0.7
Mussel	Sensitivity	0.8	0.9	1.0	1.0	0.9	1.0	1.0
	Specificity	0.9	0.8	0.7	0.7	0.7	0.7	0.7

Note: Absence of hepatitis A virus (-) in the evaluated oysters.

**Table 3:** Spearman correlation values (*r* = rho) between crAssphage and enteric viruses in oysters (*n* = 72) and mussels (*n* = 62) marketed in three cities in Rio de Janeiro, Brazil, from January to December 2022. Correlations between viral concentrations (log_10_ CG/g) were calculated using the Jamovi^®^ version 2.6.24 software.

			Viral Concentrations log_10_ GC/g						
	Bivalve	Parameters	Norovirus GII	Sapovirus	Mastadenovirus	Rotavirus A	Norovirus GI	Hepatitis A Virus	Astrovirus
CrAssphage log_10_ GC/g	Oyster	rho, *p*-level	0.024	0.227	−0.139	0.102	−0.053	-	0.389 ***
	Mussel	rho, *p*-level	0.536 ***	0.488 ***	0.537 ***	0.287 *	0.581 ***	0.252 *	0.464 ***

Note: Absence of hepatitis A virus (-) in the evaluated oysters. *** *p* < 0.001 and * *p* < 0.05.

## Data Availability

Data will be made available on request.

## References

[1] FAO (2024). Fisheries and Aquaculture Projections to 2032.

[2] Lin D., Chen W., Lin Z., Liu L., Zhang M., Yang H., Liu Z., Chen L. (2025). Viral Transmission in Sea Food Systems: Strategies for Control and Emerging Challenges. Foods.

[3] Joint FAO/WHO (2021). Technical Guidance for the Development of the Growing Area Aspects of Bivalve Mollusc Sanitation Programmes.

[4] Joint FAO/WHO (2015). Codex Alimentarius Commission. Standard for Live and Raw Bivalve Molluscs (CXS 292-2008).

[5] Ministério da Agricultura e Pecuária (MAPA) (2023). Portaria SDA/MAPA nº 884, de 6 de Setembro de 2023.

[6] Prez V.E., Gil P.M., Temprana C.F., Cuadrado P.R., Martínez L., Giordano M.O., Masachessi G., Isa M.B., Ré V., Pavan J. (2015). Quantification of Human Infection Risk Caused by Rotavirus in Surface Waters from Córdoba, Argentina. Sci. Total Environ..

[7] Sidhu J.P.S., Sena K., Hodgers L., Palmer A., Toze S. (2018). Comparative Enteric Viruses and Coliphage Removal during Wastewater Treatment Processes in a Sub-Tropical Environment. Sci. Total Environ..

[8] Yang M., Zhao F., Tong L., Wang S., Zhou D. (2021). Contamination, Bioaccumulation Mechanism, Detection, and Control of Human Norovirus in Bivalve Shellfish: A Review. Crit. Rev. Food Sci. Nutr..

[9] Desdouits M., Reynaud Y., Philippe C., Le Guyader F.S.L.G. (2023). A Comprehensive Review for the Surveillance of Human Pathogenic Microorganisms in Shellfish. Microorganisms.

[10] Joint FAO/WHO (2024). Microbiological Risk Assessment of Viruses in Foods: Part 1: Food Attribution, Analytical Methods and Indicators—Meeting Report.

[11] Lowther J.A., Cross L., Stapleton T., Gustar N.E., Walker D.I., Sills M., Treagus S., Pollington V., Lees D.N. (2019). Use of F-Specific RNA Bacteriophage to Estimate Infectious Norovirus Levels in Oysters. Food Environ. Virol..

[12] Haramoto E., Kitajima M., Kishida N., Konno Y., Katayama H., Asami M., Akiba M. (2013). Occurrence of Pepper Mild Mottle Virus in Drinking Water Sources in Japan. Appl. Environ. Microbiol..

[13] Symonds E.M., Nguyen K.H., Harwood V.J., Breitbart M. (2018). Pepper Mild Mottle Virus: A Plant Pathogen with a Greater Purpose in (Waste)Water Treatment Development and Public Health Management. Water Res..

[14] Rusiñol M., Fernandez-Cassi X., Hundesa A., Vieira C., Kern A., Eriksson I., Ziros P., Kay D., Miagostovich M., Vargha M. (2014). Application of Human and Animal Viral Microbial Source Tracking Tools in Fresh and Marine Waters from Five Different Geographical Areas. Water Res..

[15] Ballesté E., Pascual-Benito M., Martín-Díaz J., Blanch A.R., Lucena F., Muniesa M., Jofre J., García-Aljaro C. (2019). Dynamics of CrAssphage as a Human Source Tracking Marker in Potentially Faecally Polluted Environments. Water Res..

[16] Wu Z., Greaves J., Arp L., Stone D., Bibby K. (2020). Comparative Fate of CrAssphage with Culturable and Molecular Fecal Pollution Indicators during Activated Sludge Wastewater Treatment. Environ. Int..

[17] Dutilh B.E., Cassman N., McNair K., Sanchez S.E., Silva G.G.Z., Boling L., Barr J.J., Speth D.R., Seguritan V., Aziz R.K. (2014). A Highly Abundant Bacteriophage Discovered in the Unknown Sequences of Human Faecal Metagenomes. Nat. Commun..

[18] Shkoporov A.N., Khokhlova E.V., Fitzgerald C.B., Stockdale S.R., Draper L.A., Ross R.P., Hill C. (2018). ΦCrAss001 represents the most abundant bacteriophage family in the human gut and infects Bacteroides intestinalis. Nat. Commun..

[19] Heffron J., Mayer B.K. (2020). Virus Isoelectric Point Estimation: Theories and Methods. Appl. Environ. Microbiol..

[20] Farkas K., Cooper D.M., McDonald J.E., Malham S.K., de Rougemont A., Jones D.L. (2018). Seasonal and Spatial Dynamics of Enteric Viruses in Wastewater and in Riverine and Estuarine Receiving Waters. Sci. Total Environ..

[21] Stachler E., Bibby K. (2014). Metagenomic Evaluation of the Highly Abundant Human Gut Bacteriophage CrAssphage for Source Tracking of Human Fecal Pollution. Environ. Sci. Technol. Lett..

[22] Cinek O., Mazankova K., Kramna L., Odeh R., Alassaf A., Ibekwe M.U., Ahmadov G., Mekki H., Abdullah M.A., Bashir M.E. (2018). Quantitative *CrAssphage* Real-Time PCR Assay Derived from Data of Multiple Geographically Distant Populations. J. Med. Virol..

[23] Chen H., Liu C., Li Y.P., Teng Y. (2021). Integrating Metagenomic and Bayesian Analyses to Evaluate the Performance and Confidence of CrAssphage as an Indicator for Tracking Human Sewage Contamination in China. Environ. Sci. Technol..

[24] Cantelli C.P., Tavares G.C.L., Sarmento S.K., Burlandy F.M., Fumian T.M., Maranhão A.G., da Silva E.d.S.R.F., Horta M.A.P., Miagostovich M.P., Yang Z. (2024). Assessment of Gastroenteric Viruses in Marketed Bivalve Mollusks in the Tourist Cities of Rio de Janeiro, Brazil, 2022. Viruses.

[25] dos Santos N.L., Burlandy F.M., Figueiredo A.S., Lopes B.F., Villar L.M., Maranhão A.G., Salgado C.R.S., Brandão M.L.L., Miagostovich M.P., Leite J.P.G. (2025). Occurrence and Molecular Characterization of Human Astrovirus and Hepatitis a Virus in Bivalve Mollusks Marketed in Tourist Cities in Rio de Janeiro, Brazil. Food Environ. Virol..

[26] (2017). Microbiology of Food a Chain—Horizontal Method for Determination of Hepatitis A Virus and Norovirus in Food Using Real-Time RT-PCR—Part 1: Method for Quantification.

[27] Stachler E., Kelty C., Sivaganesan M., Li X., Bibby K., Shanks O.C. (2017). Quantitative CrAssphage PCR Assays for Human Fecal Pollution Measurement. Environ. Sci. Technol..

[28] Suh S.H., Lee J.S., Kim S.H., Vinjé J., Kim S.H., Park G.W. (2024). Evaluation of CrAssphages as a Potential Marker of Human Viral Contamination in Environmental Water and Fresh Leafy Greens. Front. Microbiol..

[29] Trullols E., Ruisánchez I., Rius F.X. (2004). Validation of Qualitative Analytical Methods. TrAC Trends Anal. Chem..

[30] Greenhalgh T. (1997). How to Read a Paper: Papers That Report Diagnostic or Screening Tests. BMJ.

[31] Jamovi, version 2.6.24; The Jamovi Project; [Computer Software]. https://www.jamovi.org.

[32] Farkas K., Adriaenssens E.M., Walker D.I., McDonald J.E., Malham S.K., Jones D.L. (2019). Critical Evaluation of CrAssphage as a Molecular Marker for Human-Derived Wastewater Contamination in the Aquatic Environment. Food Environ. Virol..

[33] Venuti I., Cuevas-Ferrando E., Falcó I., Girón-Guzmán I., Ceruso M., Pepe T., Sánchez G. (2025). Presence of Potentially Infectious Human Enteric Viruses and Antibiotic Resistance Genes in Mussels from the Campania Region, Italy: Implications for Consumer’s Safety. Food Environ. Virol..

[34] Gyawali P., Devane M., Scholes P., Hewitt J. (2021). Application of CrAssphage, F-RNA Phage and Pepper Mild Mottle Virus as Indicators of Human Faecal and Norovirus Contamination in Shellfish. Sci. Total Environ..

[35] Cuevas-Ferrando E., Allende A., Pérez-Cataluña A., Truchado P., Hernández N., Gil M.I., Sánchez G. (2021). Occurrence and Accumulation of Human Enteric Viruses and Phages in Process Water from the Fresh Produce Industry. Foods.

[36] Sabar M.A., Honda R., Haramoto E. (2022). CrAssphage as an Indicator of Human-Fecal Contamination in Water Environment and Virus Reduction in Wastewater Treatment. Water Res..

[37] Demoliner M., Filippi M., Gularte J.S., de Almeida P.R., da Silva M.S., Pereira V.M.d.A.G., Hansen A.W., Girardi V., Ferreira H.L., Spilki F.R. (2025). Comparison of Metagenomic Protocols for Virome Data Generation from Environmental Matrices and Stool Samples: Insights into Viral Diversity and Fecal Contamination Indicators. Total Environ. Microbiol..

[38] Liu S., Lioe T.S., Sun L., Adriaenssens E., McCarthy A., Sekar R. (2025). Validation of CrAssphage Microbial Source Tracking Markers and Comparison with Bacteroidales Markers for Detection and Quantification of Faecal Contaminations in Surface Water. Environ. Pollut..

[39] Suplicy F.M., Vianna L.F.d.N., Rupp G.S., Novaes A.L.T., Garbossa L.H.P., de Souza R.V., Guzenski J., da Costa S.W., Silva F.M., dos Santos A.A. (2015). Planning and Management for Sustainable Coastal Aquaculture Development in Santa Catarina State, South Brazil. Rev. Aquac..

[40] de Souza R.V., Moresco V., Miotto M., Souza D.S.M., de Campos C.J.A. (2022). Prevalence, Distribution and Environmental Effects on Faecal Indicator Bacteria and Pathogens of Concern in Commercial Shellfish Production Areas in a Subtropical Region of a Developing Country (Santa Catarina, Brazil). Environ. Monit. Assess..

[41] García-Aljaro C., Ballesté E., Muniesa M., Jofre J. (2017). Determination of CrAssphage in Water Samples and Applicability for Tracking Human Faecal Pollution. Microb. Biotechnol..

[42] Instituto Brasileiro de Estatística e Geografia (IBGE) (2024). Cidades e Estados do Brasil. https://cidades.ibge.gov.br/.

[43] Edwards R.A., Vega A.A., Norman H.M., Ohaeri M., Levi K., Dinsdale E.A., Cinek O., Aziz R.K., McNair K., Barr J.J. (2019). Global Phylogeography and Ancient Evolution of the Widespread Human Gut Virus CrAssphage. Nat. Microbiol..

[44] Honap T.P., Sankaranarayanan K., Schnorr S.L., Ozga A.T., Warinner C., Lewis C.M. (2020). Biogeographic Study of Human Gut-Associated CrAssphage Suggests Impacts from Industrialization and Recent Expansion. PLoS ONE.

[45] Stachler E., Akyon B., de Carvalho N.A., Ference C., Bibby K. (2018). Correlation of CrAssphage QPCR Markers with Culturable and Molecular Indicators of Human Fecal Pollution in an Impacted Urban Watershed. Environ. Sci. Technol..

[46] Nam S.J., Hu W.S., Koo O.K. (2022). Evaluation of CrAssphage as a Human-Specific Microbial Source-Tracking Marker in the Republic of Korea. Environ. Monit. Assess..

[47] Mafumo N., Bezuidt O.K.I., le Roux W., Makhalanyane T.P. (2023). CrAssphage May Be Viable Markers of Contamination in Pristine and Contaminated River Water. mSystems.

[48] Wu H., Brighton K., Chen J., Shuai D., Aw T.G. (2025). Quantification of Particle-Associated Viruses in Secondary Treated Wastewater Effluent. Food Environ. Virol..

[49] Wu H., Juel A.I., Eytcheson S., Munir M., Aw T.G., Molina M. (2023). Temporal and Spatial Relationships of CrAssphage and Enteric Viral and Bacterial Pathogens in Wastewater in North Carolina. Water Res..

[50] Karkman A., Pärnänen K., Larsson D.G.J. (2019). Fecal pollution can explain antibiotic resistance gene abundances in anthropogenically impacted environments. Nat. Commun..

[51] Ballesté E., Blanch A.R., Mendez J., Sala-comorera L., Maunula L., Monteiro S., Farnleitner A.H., Tiehm A., Jofre J., García-aljaro C. (2021). Bacteriophages Are Good Estimators of Human Viruses Present in Water. Front. Microbiol..

[52] Chen H., Bai X., Li Y., Jing L., Chen R., Teng Y. (2019). Source identification of antibiotic resistance genes in a peri-urban river using novel crAssphage marker genes and metagenomic signatures. Water Res..

[53] Crank K., Li X., North D., Ferraro G.B., Iaconelli M., Mancini P., La Rosa G., Bibby K. (2020). CrAssphage abundance and correlation with molecular viral markers in Italian wastewater. Water Res..

[54] Threndyle R.E., Kurylyk B.L., Huang Y., Johnston L.H., Jamieson R.C. (2022). CrAssphage as an indicator of groundwater-borne pollution in coastal ecosystems. Environ. Res. Commun..

[55] Ahmed W., Lobos A., Senkbeil J., Peraud J., Gallard J., Harwood V.J. (2018). Evaluation of the novel crAssphage marker for sewage pollution tracking in storm drain outfalls in Tampa, Florida. Water Res..

[56] Farkas K., Walker D.I., Adriaenssens E.M., McDonald J.E., Hillary L.S., Malham S.K., Jones D.L. (2020). Viral indicators for tracking domestic wastewater contamination in the aquatic environment. Water Res..

[57] Toribio-Avedillo D., Ballesté E., García-Aljaro C., Stange C., Tiehm A., CSánchez-Cid C., Mulogo E., Nasser A., Santos R., Nemes K. (2025). The reliability of CrAssphage in human fecal pollution detection: A cross-regional MST marker assessment. J. Environ. Manag..

